# The Role of Ultrasound Imaging in Evaluating Eagle’s Syndrome: A Case Report

**DOI:** 10.2174/0115734056352888250403063655

**Published:** 2025-04-21

**Authors:** Izim Turker Kader, Elif Celebi, Pinar Kursoglu

**Affiliations:** 1 Department of Prosthodontics, School of Dental Medicine, Bahcesehir University, Istanbul, Turkey; 2 Department of Oral and Maxillofacial Radiology, School of Dental Medicine, Bahcesehir University, Istanbul, Turkey; 3 Department of Prosthodontics, Faculty of Dentistry, Yeditepe University, Istanbul, Turkey

**Keywords:** Styloid Process, Stylocarotid Artery Syndrome, Facial Pain, Carotid Arteries, Ultrasonography, Eagle’s syndrome

## Abstract

**Background:**

Eagle’s Syndrome is a unilateral or bilateral elongation of the styloid process or calcified stylohyoid ligament, along with other symptoms, such as dysphagia, facial pain, globus sensation, and headache. Stylocarotid artery syndrome is a specific type of Eagle’s syndrome that causes various clinical symptoms due to pressure on adjacent anatomical structures.

**Case Presentation:**

This study presents a case of a 57-year-old female patient with a complaint of facial pain, head and neck discomfort, globus sensation, difficulty swallowing, and dizziness during head rotation. The patient was diagnosed with a bilateral elongated styloid process through panoramic radiography and cone beam computed tomography. Due to suspicion of stylocarotid artery syndrome, further evaluation was conducted using ultrasound imaging.

**Conclusion:**

Bilateral elongated styloid processes can contribute to Stylocarotid Artery Syndrome (SAS). Ultrasound imaging, specifically B-mode and pulsed wave Doppler, proved to be valuable in detecting real-time vascular flow dynamics in extracranial vessels, highlighting its auxiliary role in the assessment of stylocarotid artery syndrome.

## 1 INTRODUCTION

Eagle’s syndrome (ES) is an uncommon condition characterized by Styloid Process (SP) elongation or calcification of the stylohyoid ligament, leading to various symptoms [1 - 3]. The SP, a bilateral bony projection originating from the temporal bone anterior to the stylomastoid foramen, is normally 20-30 mm in length [1]. However, an abnormally long SP sometimes causes clinical symptoms due to pressure on adjacent anatomical structures, such as cranial nerves, carotid arteries, or the jugular vein [1, 4]. The symptoms of ES may include severe neck and facial pain, radiating pain to the ear usually aggravated during head rotation, swallowing, yawning, or chewing, foreign body sensation in the throat (globus sensation), odynophagia, pharyngeal paresthesia, dysphagia, dysphonia, dizziness, syncope, hypersalivation and hearing problems [1, 2, 5, 6].

Eagle’s syndrome (ES) is classified into two types [1-3]: the classical type, which presents with throat and neck pain or a foreign body sensation, and stylocarotid artery syndrome (SAS), a vascular subtype that can cause neurological symptoms, such as headache, transient ischemic episodes, or stroke due to compression or dissection of the internal carotid artery (ICA) [7].

Elongation of SP can be asymptomatic and requires imaging for diagnosis, commonly panoramic radiography, Cone Beam Computed Tomography (CBCT), and vascular assessment using CT angiography [5, 8, 9]. Ultrasonography (US) imaging, particularly with B-mode and Pulse Wave (PW) Doppler, provides a non-invasive method to evaluate real-time vascular blood flow dynamics [7, 10].

This report presents the case of a 57-year-old female with facial pain, head and neck discomfort, globus sensation, difficulty swallowing, and dizziness during head rotation. She was suspected of having SAS caused by a bilaterally elongated SP and was evaluated using panoramic radiography and CBCT.

Additionally, a B-mode and PW Doppler US imaging was conducted to evaluate the vascular flow dynamics of ICAs.

## 2 CASE REPORT

### 2.1 Patient Information

The anamnesis of a 57-year-old female patient who was admitted to our clinic revealed significance for hyperlipidemia, fibromyalgia, hypothyroidism, and gastroesophageal reflux. The patient suffered from cardiovascular diseases, including hypertension, as well as mitral and aortic valve defects due to a previous episode of acute rheumatic fever. The patient had a history of Hodgkin’s lymphoma, which was diagnosed and successfully treated with chemotherapy and radiotherapy in 1998. The patient experienced COVID-19 and cerebrovascular ischemia in 2022, which manifested as weakness on the right side of the body, loss of vision in the right eye, amnesia, and dizziness. She used various medications, including clopidogrel, atorvastatin, levothyroxine, and metoprolol.

### 2.2 Clinical Findings

The main complaint of the patient was pain in the head and neck region, with an average of 5-6 VAS scores on palpation of the masticatory muscles in the gnathological examination performed following the Diagnostic Criteria for Temporomandibular Disorders (DC/TMD). A 'click' sound was heard during mouth opening in the Left Temporomandibular Joint (TMJ). Deviation of the mandible to the right was observed when opening the mouth.

Considering these findings, the patient was diagnosed with Temporomandibular Disorders (TMDs) and bruxism during the intraoral examination. In addition to the complaints of pain in the masticatory muscles and neck regions, our patient also mentioned different problems that did not comply with TMD, including symptoms, such as globus sensation, difficulty swallowing, and dizziness when turning the head.

### 2.3 Diagnostic Assessment

**Fig. (1):**
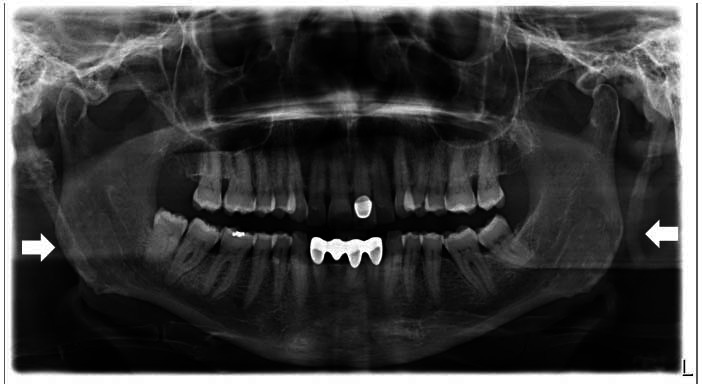
Panoramic radiograph showing the bilateral mineralized styloid processes (arrowheads).

The panoramic radiograph (Fig. **1**), requested as a primary examination method for differential diagnosis, detected longer-than-normal and calcified right and left SPs. Subsequent imaging with CBCT was performed to evaluate the length and position of the bilateral elongated SPs.

**Fig. (2a):**
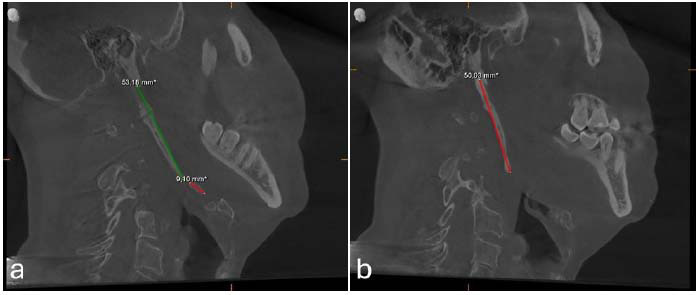
On Cone Beam Computed Tomography (CBCT) sections, the right styloid process was measured 53 mm with a segmented portion (10 mm in diameter) at the terminal edge, and (**b**) the left styloid process measured 50 mm. In the consultation with the otolaryngologist, it was thought that the signs of pain on palpation of the tonsillar fossa and the symptoms of dizziness may cause pressure on the carotid arteries of the patient's SPs and cause a change in blood flow. The patient was further evaluated by the US imaging to visualize the relationship of SPs with carotid arteries and jugular veins.

The measurement of the right SP was approximated at 53 mm in the CBCT, revealing a pseudo-articulation at the C1 level. Additionally, the CBCT on the right side exhibited a segmented portion (10 mm in diameter) at the terminal edge of the complex, situated at the lesser horn of the hyoid bone. Langlais’s classification includes three types of radiographic appearance and four patterns of calcification or mineralization: type I-elongated with a calcified outline, type II-pseudo articulated partially calcified type, and type III-segmented completely calcified or with a nodular pattern of calcification [11]. Following the classification, the right SP was identified as type II - a pseudo-articulation with a calcified outline. On the other hand, the left SP measured 50 mm and was classified as type I - elongated. The left SP displayed a medial curvature and partially calcified pattern (Fig. **2**).

**Fig. (3a):**
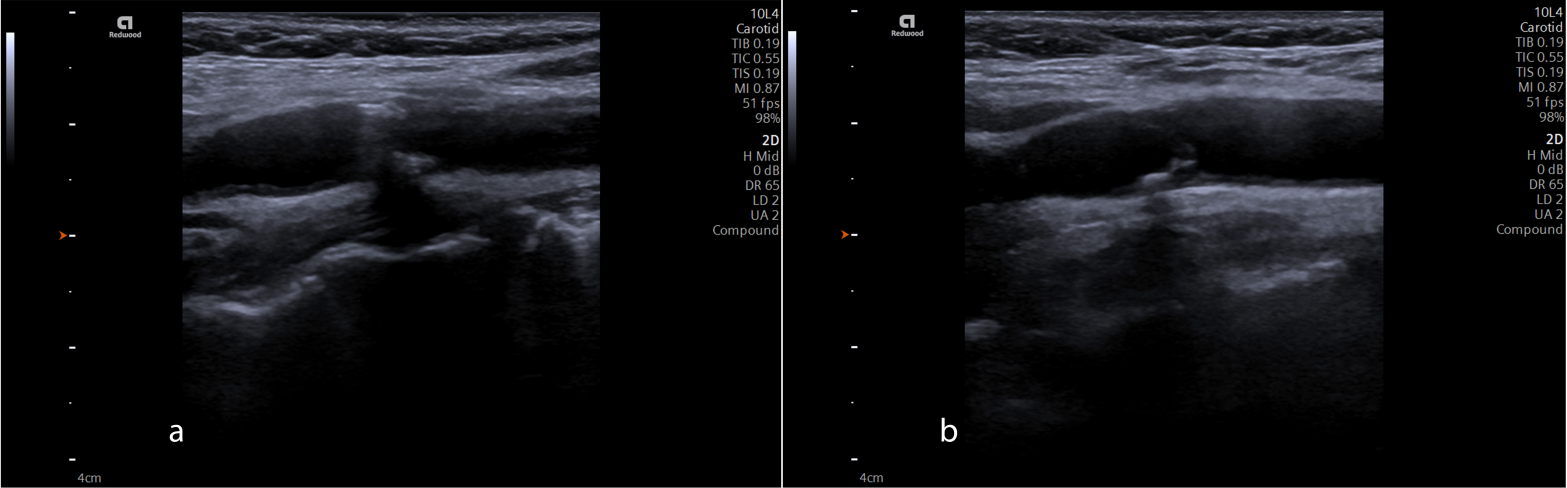
B-mode ultrasonography showed a calcific plaque accumulation and narrowing of the area on the proximal segment of the left internal carotid artery (LT PROX ICA) and (**b**) the right internal carotid artery (RT PROX ICA) in the normal position.

In the B-mode images of the proximal segment of ICAs, calcific plaque accumulation and narrowing of the area were observed in the patient (Fig. **3**). Considering the patient's cardiological history, it was suggested that calcific plaque accumulation might have altered blood flow. Given the clinical findings and the suspicion of SAS, prolonged SP-induced compression on the ICAs, combined with stenosis caused by calcific plaques, might have contributed to these hemodynamic changes. Consequently, blood flow velocity was also assessed during head rotation.

**Fig. (4):**
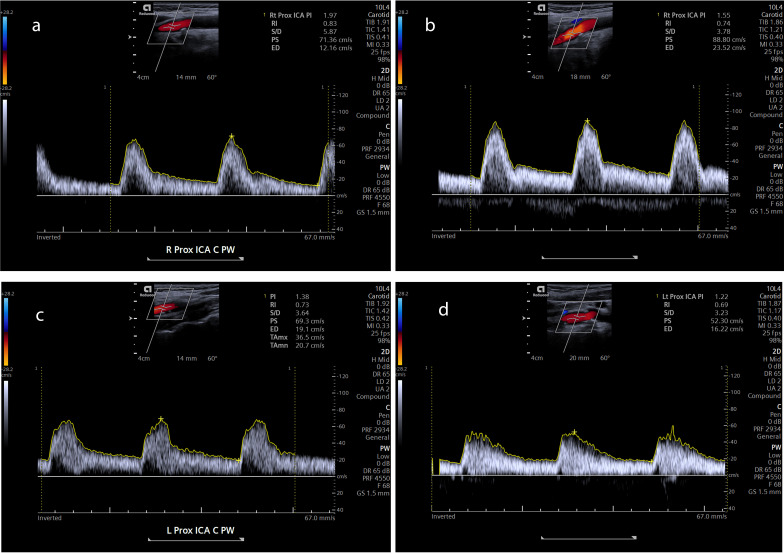
PW Doppler imaging showed the blood flow velocity of the proximal segment of the right ICA in normal position: PSV=71.36 cm/s, EDV=12.16 cm/s (**a**) The blood flow velocity in the right ICA increased when the patient slowly turned head to the left: PSV=88.80 cm/s, EDV=23.52 cm/s. (**b**) The left ICA in normal position: PSV=69.3 cm/s, EDV=19.1 cm/s (**c**) The PSV and the EDV blood flow velocity in left ICA decreased when the patient slowly turned head to the right: PSV=52.30 cm/s, EDV=16.22 cm/s. (**d**) (ICA, internal carotid artery; PSV, peak systolic velocity; EDV, end-diastolic velocity; PW, Pulsed wave).

Blood flow velocity in the right ICA increased as the patient progressively rotated her head to the left, significantly different from the normal position. When the head was rotated to the left, Pulsed wave (PW) Doppler imaging showed that the Peak Systolic Velocity (PSV) and End-Diastolic Velocity (EDV) increased. In the normal position, the blood flow velocity in the right ICA was PSV=71.36 cm/s, EDV=12.16 cm/s (Fig. **4a**); however, when the patient slowly rotated her head to the left, the blood flow velocity in the right ICA was PSV=88.80 cm/s, EDV=23.52 cm/s (Fig. **4b**). Increased blood flow velocity through the right ICA revealed that it had been compressed. Although the blood flow pattern appeared to be altered in the left ICA (Fig. **4c**, **d**), no significant changes in PSV and EDV were detected in the US imaging.

### 2.4 Treatment and Prognosis

The patient was referred to the cardiovascular diseases department to evaluate the relationship between SPs and carotid arteries during function and to assess calcific plaques in detail. A consultation determined no significant cardiovascular concern related to the increased blood flow velocity in the right ICA. Further evaluation revealed that stenosis in both the right and left ICAs was less than 70%. The current dosages of clopidogrel, atorvastatin, and metoprolol were deemed appropriate. Treatment options, including styloidectomy, were discussed, but the patient declined surgical intervention. Annual follow-up with US imaging was planned, and analgesics and muscle relaxants were prescribed for TMD and muscle pain. The patient, recently prescribed venlafaxine, was advised to maintain communication with her psychiatrist for medication management.

## 3 DISCUSSION

Recognition of ES presents a diagnostic challenge for clinicians, requiring a thorough patient history along with clinical and radiological findings [12]. The syndrome was first included in the *International Classification of Headache Disorders* (ICHD) third edition (ICHD-3) (beta version; 2013) with specific diagnostic criteria [13]. These criteria include head, neck, pharyngeal, and/or facial pain; radiological evidence of a calcified or elongated stylohyoid ligament; pain provoked or exacerbated by palpation of the stylohyoid ligament; pain triggered by head rotation; significant relief following local anesthetic injection or styloidectomy; pain localized ipsilaterally to the affected ligament; and exclusion of other ICHD-3 diagnoses.

SAS is a rare vascular type of ES [14], which is caused by ICA compression, causing facial pain exacerbated by the contralateral rotation of the head [12]. The impingement of the SP on the carotid vessels may irritate the sympathetic nerves in the arterial sheath. There have been rare cases of carotid artery dissection, cerebral ischemia, stroke, and even sudden death as attributable to prolonged SP [2, 15 - 17]. González-García *et al*. reported that SAS is not included in the ICHD-3 beta definition of ES [12]. However, it is not possible to make a complete distinction because the symptoms may be similar to each other.

CT is essential in evaluating SAS, as it detects anatomical abnormalities and identifies neurovascular conflicts [8, 9]. Three-dimensional reformatting further aids in establishing the relationship between an elongated SP and adjacent blood vessels [18]. Understanding the spatial relationship between the SP and carotid arteries is crucial for differential diagnosis. Carotid US imaging, particularly with Doppler mode, has been utilized in suspected SAS cases for screening velocity abnormalities, preoperative assessment, and surgical postoperative evaluation, as reported in case reports by Li *et al.* [7] and Esiobu *et al.* [10]. Notably, Li *et al.* [7] used US imaging to evaluate a suspected SAS case in a patient who experienced transient ischemic attacks. In the current case, bilateral elongated SPs were diagnosed through panoramic radiography and CBCT, with SAS further assessed *via* US imaging. Accelerated blood flow in the right ICA during left head rotation confirmed vascular compression by the elongated SP.

It is necessary to differentiate findings overlapping with SAS in relation to the patient's condition. Although the patient had TMD accompanied by head and neck pain, other findings point toward SAS. The differential diagnosis was made based on the additional symptoms presented. Regarding the cardiovascular status, the patient was referred to the cardiovascular department, where evaluations revealed controlled hypertension and vascular stenosis below 70%, ruling out significant atheroma-related concerns [19]. While the cardiovascular specialist suggested a link between the prior cerebrovascular ischemic event and the patient’s COVID-19 history, the role of SAS in transient ischemic attacks remains noteworthy. These findings underscore the importance of evaluating elongated SPs and their potential impact on vascular structures in suspected SAS cases.

Additional assessments excluded other potential causes, such as thromboangiitis obliterans (Buerger’s disease), giant cell arteritis, tumour-related compression or invasion of the carotid artery, or intracranial space-occupying lesions. Considering the evaluation boundaries of the US imaging, a thorough assessment of symptoms is essential for differentiation, as SAS exhibits distinct characteristics compared to these conditions (Table **1**).

Buerger’s disease primarily affects distal vessels, including small and medium-sized arteries and veins in the extremities, and is strongly linked to smoking [20]. Giant cell arteritis often presents systemic symptoms, such as fever and malaise, with the halo sign on the US imaging indicating arterial wall edema [21,22]. Tumour-related compression or invasion of the carotid artery appears as a space-occupying mass in the carotid region on B-mode and PW Doppler US imaging and can cause sudden changes in blood flow [23,24]. However, tumour presence in this region is typically confirmed using MRI or CT [23]. In the presented case, CBCT imaging and a detailed head and neck examination revealed no evidence of a tumour. Intracranial space-occupying lesions can cause symptoms like headache, painful trigeminal neuropathy, and visual disturbances resembling the presented case [25-27]. However, the presence of globus sensation, swallowing difficulties, and dizziness during head rotation suggests an alternative diagnosis, as these symptoms are not typically associated with intracranial lesions. Moreover, intracranial lesions generally do not impact ICA flow unless secondary involvement occurs [28]. US imaging plays a key role in diagnosing SAS by detecting vascular compression and flow alterations induced by styloid process elongation, distinguishing it from other conditions.

The role of the US imaging in evaluating SAS is still limited in the literature [7, 10]. However, it is a valuable, objective, and non-invasive method for evaluating SAS potentially caused by an elongated SP [7] and for confirming treatment success [10]. Unlike standard US imaging, B-mode and PW Doppler US imaging offer both structural visualizations of tissues and real-time evaluation of vascular hemodynamics, such as blood flow velocity and patterns. This is particularly beneficial in SAS cases, as it enables the detection of dynamic changes during head movements, which may not be evident in conventional imaging [7]. Further studies are necessary to establish the full potential of US imaging in SAS evaluation.

Treatment options for ES, including SAS, involve medical management, such as analgesics, corticosteroids, antidepressants, anticonvulsants, and surgical intervention, with Styloidectomy being the definitive treatment for symptomatic cases [29, 30]. In this case, the patient was informed of these options but declined surgery. Instead, annual follow-up with US imaging was planned to monitor the progression of SAS.

## CONCLUSION

This case highlights the utility of US imaging, particularly B-mode and PW doppler, in evaluating SAS associated with bilateral elongated SPs. While panoramic radiography and CBCT confirmed the structural elongation, US imaging provided a crucial real-time assessment of vascular flow dynamics, revealing carotid artery involvement during head rotation. This underscores US imaging as a non-invasive and complementary diagnostic tool for SAS and a means to monitor its progression. Moreover, a multidisciplinary approach, including consultations with specialists from various fields, is essential to evaluate the necessity of intervention and ensure comprehensive patient care.

## Figures and Tables

**Table 1:** Ultrasonographic evaluation for the differential diagnosis of carotid artery compression.

**Ultrasonographic (US) evaluation**	**Eagle’s syndrome (SAS type)**	**Thromboangiitis obliterans (Buerger’s disease)**	**Giant cell arteritis**	**Tumour-related compression or invasion of carotid arteries**	**Intracranial space occupying lesions**
**Symptoms**	Severe neck and facial pain, radiating pain to the ear usually aggravated during head rotation, swallowing, yawning, or chewing, foreign body sensation in the throat (Globus sensation), odynophagia, pharyngeal paresthesia, dysphagia, dysphonia, dizziness, syncope, hypersalivation, and hearing problems [1, 2, 5, 6]	Calf or foot claudication, ischemic pain at rest and ischemic ulcerations on the toes, feet, or fingers [20]	-Polymyalgia rheumatica represented by fever, pain in the shoulders and hips, malaise, and weight loss. -Medium temporal headache -Claudication of the masticatory and tongue muscles [21]	-Painless and palpable neck mass -Local pressure of the artery represented by neck or ear pain, sore throat, local tenderness, and odynophagia -Dysphasia, dysarthria, swallowing difficulties, Horner's syndrome, hoarseness, dizziness, headache, flushing, palpitations, tachycardia, arrhythmia, diaphoresis and photophobia [23]	-Asymptomatic lesions -Headache, painful trigeminal neuropathy, visual disturbances, seizures, increased intracranial pressure, dementia, hemiparesis, vomiting, aseptic chemical meningitis, focal neurological signs, altered consciousness, and hydrocephalus [25,26]
**B-mode US**	Real-time assessment of carotid artery blood flow dynamics, including evaluation of calcific plaque accumulation and arterial narrowing. [7,10]	The normal structure of the arterial wall typically remains intact [20]	Arterial wall thickening, dark halo sign (arterial wall edema) [21]	Visible solid mass, vessel displacement or compression due to mass effect [23]	Detection of the lesion location using intracranial US imaging [27]
**CDFI/PW Doppler US**	Evaluation of real-time hemodynamic changes, including blood flow velocity alterations due to styloid process compression on the ICAs during head rotation [7,10]	Non-atherosclerotic vascular occlusion, collateral vessel formation, and decreased blood flow [20]	Stenosis and altered blood flow velocities due to segmental arterial inflammation lead to discontinuous vessel involvement and hourglass-shaped occlusion [21,22]	Hypervascularization within the tumour [23] Sudden changes in blood flow [24]	Altered intracranial blood flow patterns due to secondary involvement within the apparent mass [28], enabling lesion detection and vascular visualization via intracranial US [27]
**Characteristics**	ES can be identified using panoramic radiography and CBCT, while SAS can be further evaluated with ultrasound imaging. Hemodynamic changes in the ICAs during head rotation can confirm vascular compression by the elongated SP. [7,10]	-Buerger’s disease primarily affects the distal arteries and upper limbs, distinguishing it from atherosclerotic arteriopathies. -Strongly associated with smoking, its diagnosis requires correlating clinical assessment with US imaging findings due to its atypical vascular distribution. [20]	Local ischemia, segmental arterial narrowing due to wall thickening, leading to stenosis or occlusion, non-atherosclerotic vascular obstruction, and elevated inflammatory markers. While imaging techniques vary depending on the affected artery, it is generally evaluated using US imaging, MRI, CT, and PET-CT. [21]	Although the imaging method varies based on tumour location, the presence of a tumour in the carotid region is often confirmed with MRI or CT. Vascular involvement and blood flow alterations caused by the tumour can be assessed using US imaging [23]	ICAs are usually normal, though indirect signs may be present. In addition to the US imaging, diagnosis requires MRI or CT, often correlated with focal neurological deficits. [27]

**Abbreviations**CBCT; Cone beam computed tomography, CDFI; Color Doppler flow imaging, CT; computed tomography, ICA; Internal carotid artery, MRI; Magnetic resonance imaging, PET-CT; Positron emission tomography-computed tomography, PW; Pulsed-wave, SAS; Stylocarotid artery syndrome, US; Ultrasound.

## Data Availability

The data and supportive information are available within the article from the corresponding author [İ.T.K] on reasonable request.

## LIST OF ABBREVIATIONS

SAS: Stylocarotid Artery Syndrome
ES: Eagle’s syndrome
SP: Styloid Process
ICA: Internal carotid artery
CBCT: Cone Beam Computed Tomography
PW: Pulse Wave
DC/TMD: Diagnostic Criteria for Temporomandibular Disorders
TMJ: Temporomandibular Joint
TMDs: Temporomandibular Disorders
PSV: Peak Systolic Velocity
EDV: End-Diastolic Velocity
PW: Pulsed wave

## STANDARDS OF REPORTING

CARE guidelines were followed.
